# Teaching and communicating science in a digital age

**DOI:** 10.12688/f1000research.6323.1

**Published:** 2015-03-31

**Authors:** Graham Scott

**Affiliations:** 1 School of Biological, Biomedical and Environmental Sciences, University of Hull, Hull, HU6 7RX, UK

**Keywords:** digital age, education, science communication

## Abstract

We assume that digital literacy and access are common to all who teach and communicate their science and to their audiences. We also assume that our digital communication is effective and that by using digital technologies learning experiences are enhanced. But are these reasonable assumptions to make? This *F1000Research* channel brings together papers developed from presentations made at *Teaching and Communicating Science in a Digital Age*, a Society for Experimental Biology symposium involving Higher Education Professionals from across the globe to reflect upon the impact that digital technologies have and will have upon aspects of the communication of science. Here I share some thoughts on the discussion that took place and on the papers collated through this channel.

## Editorial

Although we have come to take for granted that we live and work in a digital age we are in reality only at the beginning of our digital journey. This *F1000Research* channel brings together papers presented at *Teaching and Communicating Science in a Digital Age*, a Society for Experimental Biology symposium (15 ^th^–17 ^th^ December, 2014, London UK) that brought together Higher Education Professionals from more than 10 countries and 5 continents. Held one day after the 20th anniversary of the launching of the first graphical web browser *Netscape Navigator*, the symposium invited participants to discuss the impact and possibilities for the communication of science brought about by the on-going digital revolution.

Key amongst the themes that came to the fore during the course of the symposium and that are captured in the papers collated in this channel is the observation that to be effective digital technologies must be used alongside effective pedagogies to enhance communication in a teaching context. In her plenary Gleadow (2014) captured this idea perfectly when she explained that perhaps *evolution* rather than *revolution* best described the impact of digital technologies in teaching. Through social media, students and their tutors are able to engage in a new way – the learning conversation has become more egalitarian and has moved out of the classroom. Students blog and tweet and by doing so can enter into a new discourse with scholars and the wider public beyond their home institution in a way that would not have been imagined at the end of the twentieth century. Maker Education ( Sharples *et al.*, 2013), and creative practices such as digital filmmaking are providing new ways for students to engage with and express their learning and even within the traditional context of a large group lecture students are actively engaging with one another and with their tutor through portable devices, smartphones and clickers.

Although the reach of the Internet is global the ability to effectively receive it is not. The symposium reflected upon the fact that for many there exist political, cultural infra-structural and language barriers that limit access to digitally delivered resources and digital interaction. But as a counter point we also heard about MOOCs (Massive Open Online Courses) that enable a global community to engage with global issues and about the role of learned societies in the production of open access resources in multiple languages that can be used by students, teachers and policy makers alike in ways that may not have been imagined by their authors.

Excitingly, and perhaps uniquely, this channel also includes a paper by Hubbard *et al.*, (2015) that rather than being the result of a presentation made at the meeting is in fact a new collaboration that is the result of their individual responses to a presented paper and to the closing plenary in which delegates were asked to share perspectives on the future of education. Taken together these papers suggest that as a community of educators in Higher Education the evolution of digital communication in education has brought us to a place where if we are to fully capitalise on the opportunities available to us, we need to reflect upon the support provided to those scientists who have a teaching/engagement focus at all career stages.
